# Overall Survival Signature of 5-Methylcytosine Regulators Related Long Non-Coding RNA in Hepatocellular Carcinoma

**DOI:** 10.3389/fonc.2022.884377

**Published:** 2022-05-24

**Authors:** Qi Pan, Caiyu Yi, Yijie Zhang

**Affiliations:** ^1^ Key Laboratory of Organ Transplantation of Liaoning Province, Department of Hepatobiliary Surgery and Organ Transplantation, First Hospital of China Medical University, Shenyang, China; ^2^ China Medical University, Shenyang, China

**Keywords:** 5-methylcytosine RNA methyltransferases, long non-coding RNA, weighted gene co-expression network analysis (WGCNA), liver hepatocellular carcinoma, prognosis model

## Abstract

**Purpose:**

Studies reported that 5-methylcytosine (m5C) RNA transferase alters tumor progression; however, studies of m5C-related lncRNA remain lacking. This article intends to study the lncRNA modified by m5C RNA transferase in hepatocellular carcinoma using a combination of computational biology and basic experiments.

**Method:**

We identified 13 m5C RNA transferase-related genes and selected long non-coding RNAs with a Pearson correlation coefficient greater than 0.4. Univariate Cox regression analysis was used to screen m5C RNA transferase lncRNA related to survival phenotype. We divided TCGA-LIHC into two types of m5C RNA using non-negative matrix decomposition. According to WGCNA, the co-expression models of two lncRNA regulation modes were constructed to analyze the characteristic biological processes of the two m5C RNA transferase-related lncRNA gene models. Then, a predictive model of m5C RNA transferase lncRNA was using LASSO regression. Finally, we used cell experiments, transwell experiments, and clone formation experiments to test the relationship between SNHG4 and tumor cell proliferation in Hep-G2 and Hep-3b cells line.

**Results:**

We identified 436 m5C RNA transferase-related lncRNAs. Using univariate Cox regression analysis, 43 prognostic-related lncRNAs were determined according to P < 0.001. We divided TCGA-LIHC into two regulation modes of m5C RNA transferase using non-negative matrix factorization. The two regulation modes showed significant differences in overall and disease-free survival. We used LASSO to construct m5c-related lncRNA prognostic signature. Thus, a predictive m5C-lncRNA model was established using four lncRNAs: AC026412.3, AC010969.2, SNHG4, and AP003392.5. The score calculated by the m5C-lncRNA model significantly correlated with the overall survival of hepatocellular carcinoma. The receiver operating characteristic curve and decision curve analysis verified the accuracy of the predictive model. We observed a more robust immune response in the high-risk score group. The transwell experiments and clone formation experiments suggested that m5C RNA transferase-related lncRNA SNHG4 promotes the proliferation and migration of Hep-G2 and Hep-3b cells line.

**Conclusion:**

Two lncRNA expression patterns regulated by m5C RNA transferase were identified. The difference between the two expression patterns and the survival phenotype in the biological process was pointed out. A 5-methylcytosine RNA methyltransferases-related lncRNA overall survival signature was constructed. These results provide some understanding of the influence of m5C transferase on hepatocellular carcinoma. The prediction model of m5C transferase lncRNA has potential clinical value in managing hepatocellular carcinoma.

## Introduction

Hepatocellular cancer (HCC) is the sixth most common cause of malignant tumors. In 2020, there will be 900,000 new cases of stem cell cancer worldwide, making HCC the third leading cause of tumor-related death worldwide (1). HCC accounts for nearly 90% of primary liver cancers (2). Because the initial symptoms of HCC are not apparent, many patients are diagnosed with advanced liver cancer, hampering the success of treatment. In recent years, chemoradiotherapy for HCC has benefited patients with progressive disease; however, some patients remain with poor outcomes. Therefore, predicting the outcome of HCC patients by gene sequencing technology can assist clinicians in diagnosis and treatment strategies.

High heterogeneity is a significant feature of HCC. The primary characteristics of high heterogeneity are multiple genomic alterations and epigenetic modifications. Of these, epigenetic modifications are closely associated with tumor progression and metastasis and can be used as targets for cancer treatment. Epigenetics consists of the modification of DNA, RNA, and protein levels. Compared with the relatively limited spectrum of DNA modifications (six types), the abundance of RNA modifications is much higher. Post-transcriptional modification of RNA is an area of intense study. Of the 170 post-transcriptional modifications of RNA discovered to date, 2/3 are methylation modifications, including m1A, m6A, m5C, and m7G (3). Methylation of RNA 5-methylcytosine (m5C) is methylation at the fifth carbon atom of an RNA cytosine. This modification was discovered in rRNA in the 1970s and then successively in transport RNA, messenger RNA, and long non-coding RNA (lncRNA). M5C modification of RNA exists widely in cells and plays an essential role in regulating gene expression and RNA stability. In addition, m5C methylation is associated with proto-oncogene activation, and m5C modified methyltransferase NSUN2 is differentially expressed in tumor and para cancer tissues.

LncRNA is defined as a DNA transcript with no coding protein action over 200 bp in length (4), first proposed in a study of mouse cDNA sequencing (5). LncRNA is classified as lncRNA, antisense lncRNA, bidirectional lncRNA, intragenic lncRNA, and intergenic lncRNA, depending on its location in the genome (6). RNA methylation of lncRNA has been demonstrated in cancer progression. For example, in HCC, the m6A “writer” METTL3 increases the stability of LINC00958 and promotes cancer progression (7). Similarly, m6A “eraser” ALKBH5 increases the invasion and metastasis of gastric cancer tumor cells by inhibiting the methylation of NEAT1 (8).

In the present study, we analyze 5mC RNA methyltransferase-related lncRNA using computational biology and basic experiments to provide a basis for studying the heterogeneity of HCC.

## Methods

### Expression Collection

The gene transcripts and clinical features of the tumor tissues of patients with HCC were obtained from TCGA (https://cancergenome.nih.gov/), including 374 samples of HCC tissues and 50 samples of normal adjacent tissues. The clinical characteristics of patients included gender, survival status, survival time, tumor stage, and TNM stage.

### Screening for Differential m5C-Related lncRNA

The “EdgeR” program package in RStudio software used applied, and “FDR <0. 1, | log2FC |> 2” was the standard initially to screen the differentially expressed m5C related lncRNA. The “DEseq2” program package was used to identify differentially expressed m5C-related lncRNA according to “Padj < 0.05 and |log2FC| > 2.”

### Negative Matrix Factorization (NMF) Clustering of m5C Related lncRNAs Gene Sets

Thirteen m5C-related genes were collected from literature mining (9–20). Based on Pearson coefficient >0.4 and cox coefficient P<0.001. The m5C related lncRNAs were uploaded as **Supplementary Table 1**—the 43 m5C related lncRNA genes for non-negative matrix dimensionality reduction clustering NMF. The non-negative matrix dimensionality reduction method was implemented using the “NMF” R package (21).

### Weighted Correlation Network Analysis

A weighted standard expression network was constructed using the R language WGCNA package (22). The pickSoftThreshold function was used to obtain the optimal value of weighting parameters of adjacent parts, which was used as a soft threshold for subsequent network construction. Then, the weighted adjacency matrix was then constructed, and the related gene modules were built using hierarchical clustering based on the dissimilarity measure (1-Tom) of the topological overlap matrix (23). To determine the biological significance of each module, the potential correlation between genes and clinical traits was calculated using the characteristic genes of each module as the main component, and the expression patterns of genes of each module were summarized. Then, the correlation between the module significance and the average gene significance within the module was calculated. Finally, the correlation between the co-expression module and the expression pattern of NMF clustering subtypes was calculated.

### LASSO Regression

The LASSO (24) regression algorithm was used to identify genes related to the outcome and survival of hepatocellular cancer patients and construct a risk-scoring model. The model’s predictive performance was evaluated by the time-dependent receiver operating characteristic curve (ROC). Kaplan-Meier survival curves were used to compare survival differences of HCC patients between the two groups using the log-rank test.

### GSEA

We used GSEA 4.1.0 software with the c2.cp.kegg.v7.0.symbols.gmt dataset in the Molecular Signature Database as the functional gene set to perform GSEA for patients in different risk groups (25). The iterative operations were set to 1000, and other parameters were set to default values.

### The Proportion of Infiltrating Immune Cells in HCC

We used six methods to evaluate the relative proportion of immune infiltrating cells in the immune microenvironment, namely CIBERSORT (26, 27), EPIC (28), quanTIseq (29), MCPcounter (30), XCELL (31), and TIMER (32) algorithms to evaluate the immune response of different risk scores. We used Heatmap to analyze the differences in immune responses using the various algorithms.

The Estimation of Stromal and Immune cells in Malignant Tumor tissues using Expression data (ESTIMATE) is an algorithmic tool. The detailed algorithm is shown in **Supplementary File 2**.

### The Correlation Between Risk Score and Immune Inflammation Response

We selected several classic immune-related sub-gene sets, including primary histocompatibility complex class II, lymphocyte-specific kinase, hematopoietic cell kinase, immunoglobulin G, signal transduction, and activation transcription 1, costimulatory molecule, interferon, and TNF gene sets (33). Genes with concentrations are displayed in **Supplementary Table 3**. We analyzed the association between risk scores and the genes associated with immune responses.

### Cell Culture

The Hep-G2 and Hep-3b cell line was provided by the Shanghai Cell Bank of the Chinese Academy of Sciences. Cells were cultured in a complete DMEM medium containing 10% fetal bovine serum and placed in an incubator at 37°C and 5% CO_2_. Cells were seeded in 6-well plates at 4×10^5^ cells per well, and we observed cell fusion after culturing overnight for subsequent experiments.

### Cell Transfection Experiment

We selected Hep-G2 and Hep-3b cells in the logarithmic growth phase, trypsinized them, and seeded them in 6-well plates. After adherence, according to the lentivirus packaging manual, we transfected the cells with a multiplicity of infection of 10. After 24 hours, we added two μl of polybrene at a final concentration of 5 μg/ml for screening for 1–2 weeks, incubated at 37°C, and changed the medium once according to cell status 8–12 hours. We transferred the successfully transfected cells from each group to the cell flask and continued culturing to obtain stable cells. Cells were grouped as follows: Si-NC group, si1-SNHG4 group, and si2-SNHG4 group. Si-SNHG4F: GTCAGCGAGCGAACCCAATTGGC; R: CCGATCGGCAGCCGCGCGCGA.

### RT-PCR Detection of SNHG4 Gene Expression

We extracted the total RNA from each group of cells after transfection and reverse transcribed the RNA into cDNA according to kit instructions. We designed the primer sequence and used the cDNA containing the amplified sequence as a template for PCR reaction. After the response, the results of each group were recorded, and GAPDH was used as an internal control to compare and analyze the expression of SNHG4 in each group. SNHG4 F: CCGCCGATAGGAGCGACACCCCAAC, R: AACCATCGAGCGGGGGCTCTCGCAAA.

### Clone Formation Experiment to Observe the Effect of SNHG4 Gene on the Proliferation of Hep-G2 and Hep-3b Tumor Cell Line

After the cells were transfected, we transferred cell suspensions to 1.5 mL Eppendorf tubes, mixed and diluted, and inoculated 6-well plates at 20,000/well. We changed the medium once every three days and cloned for about ten days to observe the formation of cloning groups. The medium was then aspirated, and the cells were washed and fixed in 4% paraformaldehyde for 15 min, followed by staining with 0.1% crystal violet for 15 min. Finally, cells were washed, dried, and photographed to count clonal cell clusters and perform statistical analysis.

### Transwell Method to Observe the Effect of SNHG4 Gene on the Migration of Hep-G2 and Hep-3b Cells

We added 200 μL of HepG2 cell line suspension (1×10^4^ cells) to the upper chamber of the Transwell chambers. The experiment was divided into regular cell group (si-NC), SNHG4 gene knockdown 1 group (si1-SNHG4), and SNHG4 gene knockdown 2 Group (si2-SNHG4). Cells were placed in a 37 °C incubator for 24 hours, after which the upper chamber was removed and washed with PBS three times. Cells were then fixed with paraformaldehyde for 20 minutes, stained with 0.1% crystal violet for 30 minutes after air-drying, and we randomly selected five fields under the microscope for counting. The number of cells and the ratio of the number of penetrating cells between the experimental and control groups represent cell migration changes. The Hep-3b cells line was tested using the HepG2 cell line.

### Western Blotting

First, we used precooled RIPA buffer containing protease inhibitor (Thermo Scientific, USA) (Beyotime, Shanghai, China) to extract total protein from cells. Equivalent amounts of protein samples were isolated with 4-12% SDS-Page (GenScript, Nanjing, China) and then transferred to 0.45μm PVDF membrane (Millipore, USA). The membrane was sealed with TBST containing 5% skim milk for two h and incubated with primary antibody at four °C overnight. After washing with TBST 3 times, the antibody was coupled with HRP and incubated for one h at room temperature. Immunoblots were detected by an imaging system (Bio-Rad, USA) using an enhanced chemiluminescence detection kit (Servicebio, Wuhan, China). Western blots were performed using an imaging system (Bio-RAD, USA) using an enhanced chemiluminescence assay kit (Servicebio, Wuhan, China). The primary antibodies consisted of beta-catenin (Proteintech, 51067-2-AP), cyclin D1 (Cell Signaling Technology, 55506S), and GAPDH (Cell Signaling Technology, 5174S). The above antibodies are used in accordance with manufacturer’s agreement and instructions

## Results

### The Molecular Subtypes of lncRNA Regulated by m5C in HCC Based on the NMF Classification Method

**Figure 1:**
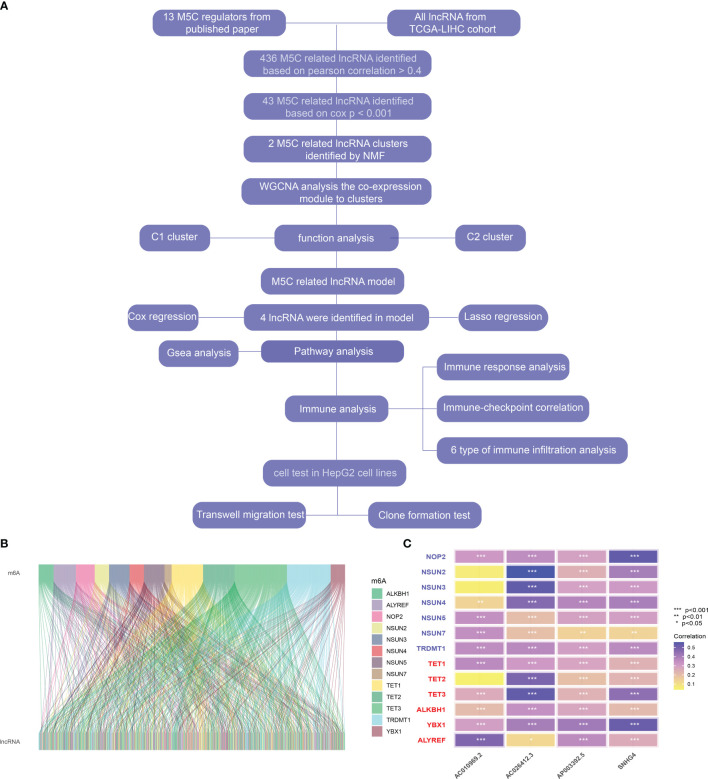
**(A)** Flow chart of this study. **(B)** Correlation between m5C correlated genes and lncRNA in hepatocellular carcinoma. **(C)** Heatmap of correlation between m5C-related genes and four prognostic m5C-related lncRNAs * represents p value < 0.05, ** represents p value < 0.01, *** represents p value < 0.001, ns means not statistically significant.

**Figure 2:**
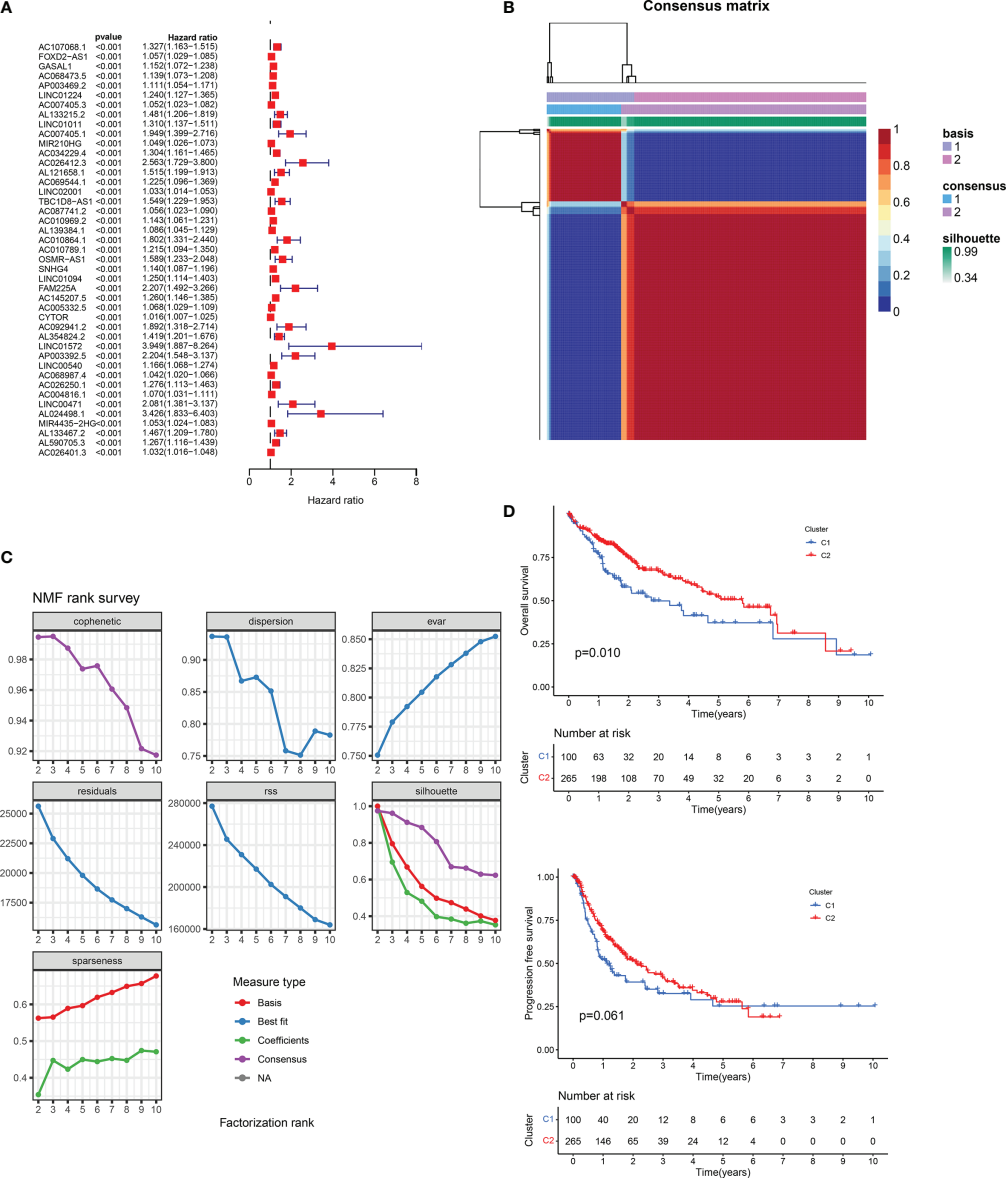
**(A)** Univariate Cox regression analysis of prognostic m5C-related lncRNAs. **(B)** Consensus map of non-negative matrix factorization clustering. **(C)** Consensus clustering parameter. **(D)** Overall survival and disease-free survival prognostic survival curves of two molecular subtypes. NA, Not application.

The flow chart of the article is shown in **Figure 1A**. The coxph function in R was used to evaluate the predictive value of m5C-regulated lncRNA. According to the Pearson correlation coefficient greater than 0.4, we identified 436 m5C-related lncRNAs (**Figures 1B, C** **)**. Then, according to the standard of single-factor Cox regression P < 0.01, we obtained 436 cancer outcome-related m5C-regulated lncRNA genes. The significance and risk ratio of m5C-regulated lncRNA significant genes are shown in **Figure 2A**. We then performed non-NMF on these prognostic-related hepatocellular cancer lncRNA-related genes using 50 iterations. We conducted nine clusters; the number of collections k was 2-10, and the minimum sample of each group was set to 10 using the ‘NMF’ R package. According to three parameters (cophenetic, dispersion, and silhouette), we choose the ideal cluster group to be 2 **(** **Figures 2B, C** **)**. We found that patients with different lncRNA gene expression patterns showed differences in overall survival and disease survival rates (**Figure 2D**; log-rank p = 0.01).

### WGCNA Gene Co-Expression Network Analysis to Identify the Biological Characteristics of Different lncRNA Groups

**Figure 3:**
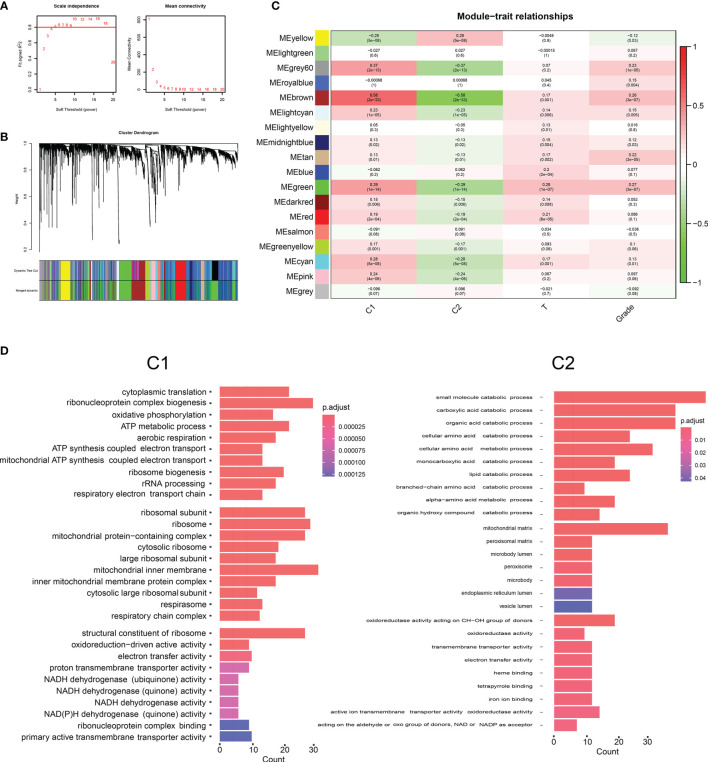
**(A)** Network topology analysis for different soft threshold powers. **(B)** A hierarchical clustering tree was constructed, with each leaf representing a gene and each branch representing a co-expression module. A total of 18 co-expression modules were generated. **(C)** The correlation coefficients between two molecular phenotypes, T stages, grade, and co-expression modules. **(D)** The primary enrichment biological pathways of co-expression modules of two molecular types.

We included the protein-coding genes and clinical samples in HCC into a WGCNA input file. In the subsequent investigation, we followed the omics cluster analysis to include samples with similar expression patterns. According to the cut-off value of 10000 and the β-value setting at 5, the gene in the smallest module is set to 30, and 18 co-expression modules are finally obtained (**Figures 3A, B**). The C1 lncRNA feature group strongly correlates with the brown module (**Figure 3C**; Cor = 0.58). We enriched the genes in the brown module that were associated with greater than 0.4 with the C1 group and found that the genes in the brown module were involved in the biological processes of oxidative phosphorylation and ATP metabolic process (**Figure 3D**). The lncRNA feature group of the C2 group had the strongest correlation with the yellow module (**Figure 3C**; Cor = 0.28). We enriched the genes in the yellow module that correlate greater than 0.4 with the C2 group. We found that yellow genes in the module were involved in the small molecule catabolic process, carboxylic acid catabolic process, and cellular amino acid metabolic process (**Figure 3D**).

### Construction of HCC Outcome Model Based on lncRNA-Related Prognostic Genes

**Figure 4:**
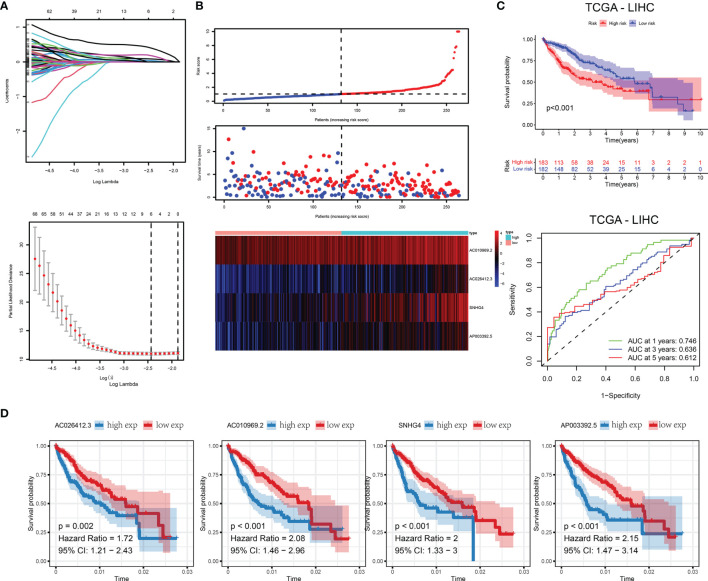
**(A)** Trajectories for each independent variable and confidence intervals for different values. **(B)** Distribution of RiskScore and survival status of 4-gene signature. **(C)** In the training set, the survival curve of the four-gene signature and the ROC curves of 1, 3, and 5 years. **(D)** Kaplan-Meier survival curves of four genes.

First, we randomly divided the entire TCGA-LIHC queue into training and validation sets. We arranged them in ascending order according to the ID of the sample and used SPSS to assign a random number to each sample for classification. The classification results satisfy the following criteria: 1) the two groups were similar in age distribution, clinical staging, follow-up time, and patient mortality; and 2) the gene expression profiles of the two randomized data sets were similar. Then we used LASSO regression to construct the lncRNA-related outcome model. First, we used the 43 prognostic-related m5C-related lncRNAs obtained above as input data and regression based on the overall survival rate as clinical follow-up data. This number of genes is not conducive to clinical detection. Therefore, to reduce the range of m5C-related lncRNAs while maintaining high accuracy, the R package glmnet was used to perform LASSO regression analysis with the trajectory of each independent variable (**Figure 4A** **)**. As the lambda increased, the independent coefficients also gradually increased, and the same was obtained for the independent coefficients. Three-fold cross-validation was used to build the model and analyze the confidence interval under each lambda. Finally, we constructed a predictive risk model containing the four genes. RiskScore = 0.75 * expAC026412.3 + 0.13 * expAC010969.2 + 0.15 * expSNHG4 + 0.33 * expAP003392.5 We calculated the RiskScore according to the expression level of the gene, and obtained the RiskScore distribution of the sample (**Figure 4B** **)**. The death rate of the high-risk samples was significantly higher than that of the samples with a low-risk score, indicating that the samples with high RiskScore had a worse outcome. We divided the RiskScore into high- and low-risk groups and drew Kaplan-Meier curves; there was a significant difference between the two **(** **Figure 4C** **)**. We used ROC to classify RiskScore. We analyzed the 1.3 and 5-year forecast classification efficiency. The 5-year AUC area was 0.612, the 3-year AUC area was 0.636, and the 1-year AUC area was 0.746. Finally, the variables in the model were used as independent prognostic factors to assess patient risk (**Figure 4D**).

### Evaluation of Model Results

**Figure 5:**
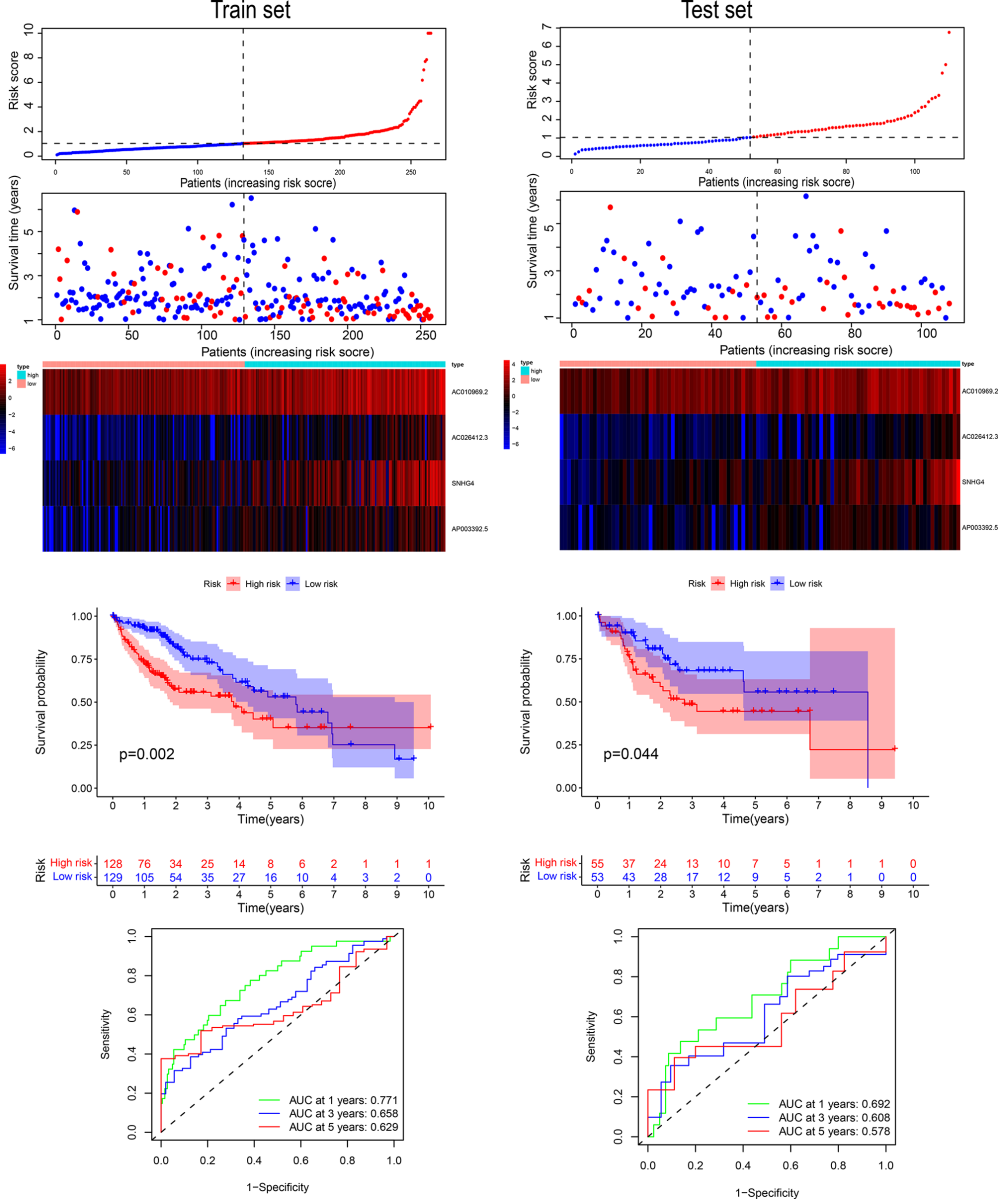
The four-gene signature’s risk score distribution and survival status in the training and validation sets. ROC curves of 1, 3, and 5 years, and survival curves between the two risk groups based on the four-gene signature classification.

**Figure 6:**
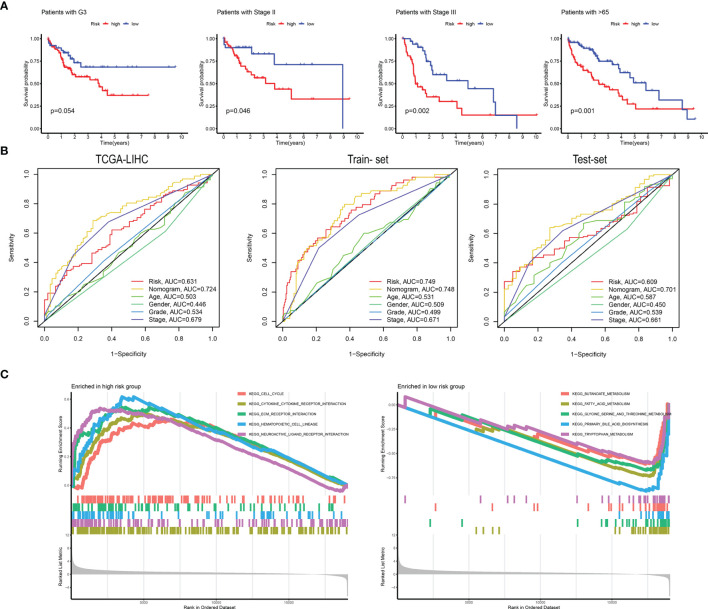
**(A)** The high-risk and low-risk groups were divided according to G3, Stage II, Stage III, and 65 years. Survival curves were plotted. **(B)** ROC curves are grouped by risk, nomogram, age, sex, grade, and stage in TCGA-LIHC, training set, and validation set. **(C)** The main KEGG pathways are enriched in the high- and low-risk groups.

We drew Kaplan-Meier curves for risk scores in the training and validation sets and found a significant difference between the high- and low-risk groups in the training and validation sets (**Figure 5**). We analyzed the prediction classification efficiency of risk scores 1, 3, and 5 years in the training and validation sets (**Figure 5** **)**. The 5-year AUC area in the training set was 0.629, the 3-year AUC area was 0.658, and the 1-year AUC area was 0.771. In the verification set, the 5-year AUC area was 0.578, the 3-year AUC area was 0.608, and the 1-year AUC area was 0.692. We included risk scores into different subgroups, such as age, stage, and others. We grouped them according to subgroup indicators to evaluate the prognostic assessment ability of risk scores in the various subgroups (**Figure 6A** **)**. The risk score distinguished patients with different outcomes in the whole cohort and patients in groups with characteristics such as age, stage, and others **(** **Figure 6A** **)**. We then compared the area under the AUC curve of the nomogram, RiskScore, age, and staging and found that the area under the curve of the risk score in the training set was the largest, with the AUC area in the training set 0.749 **(** **Figure 6B** **)**.

### GSEA Analysis

We performed GSEA analysis in high- and low-risk patients to determine pathways related to the patient’s prognostic risk. As shown in **Figure 6C**, in patients with high-risk scores of HCC, cell cycle, cytokine-cytokine-receptor interaction, ECM receptor interaction, and other tumor-related pathways were enriched. In patients with low-risk scores of HCC, butanoate metabolism, fatty-acid metabolism, and tryptophan metabolism were enriched in several tumor metabolism-related pathways.

### The Relationship Between Risk Score and Immune Microenvironment

**Figure 7:**
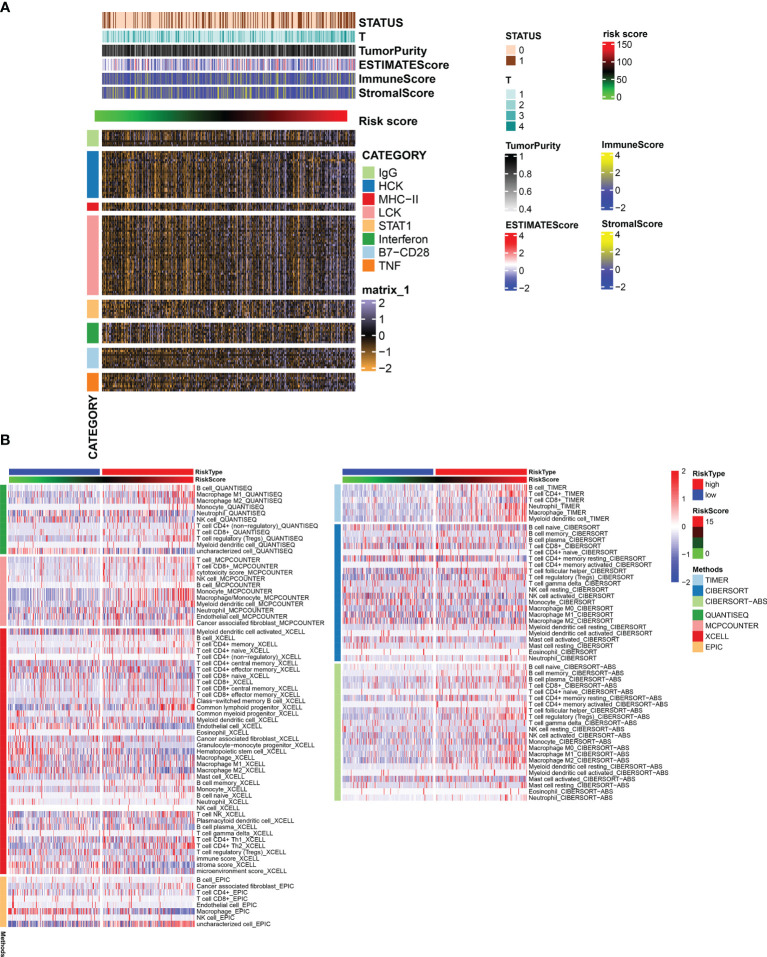
**(A)** Heatmap for genesets associated with immune and inflammation. **(B)** Heatmap for immune responses based on TIMER, CIBERSORT, CIBERSORTE-ABS, QUANTISEQ, MCPCOUNTER, XCELL, and EPIC algorithms among high- and low-risk groups.

The risk score positively correlated with inflammatory and immune responses. These reactions were induced by hematopoietic cell kinases, immunoglobulin G, interferon, lymphocyte-specific kinase, primary histocompatibility complex class I, major histocompatibility complex class II, and activator of transcription 1. Patients with higher risk scores had more clustered immune-inflammatory responses (**Figure 7A**).

### The Relationship Between Risk Score and Immune Infiltration

The relationship between the level of immune cell infiltration and risk score evaluated based on the six methods of CIBERSORT, EPIC, quanTIseq, MCPcounter, XCELL, and TIMER is shown in **Figure 7B**. We found significant differences in the level of infiltration of macrophages and CD8^+^ T cells in different RiskScore groups.

### The Effect of Knocking Out the SNHG4 Gene on the Clone Formation Ability of Hep G2 and Hep-3b Cells

**Figure 8:**
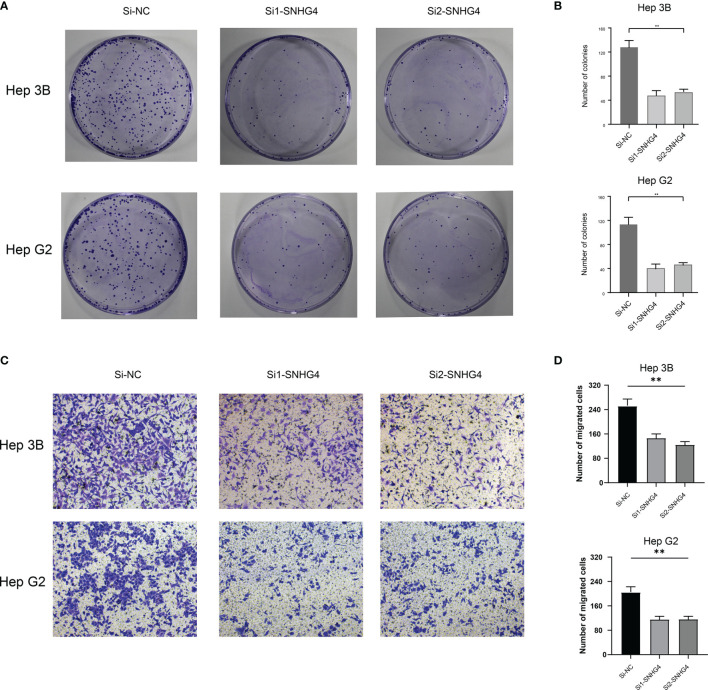
**(A, B)** Clone formation assay. **(C, D)** Transwell assay. * represents p < 0.05; ** represents p < 0.01; *** represents p < 0.001.

The above paper constructed a predictive scoring model based on M5C methylation-related long non-coding RNA. The predictive scoring gene model contained four long non-coding RNAs: AC026412.3, AC010969.2, SNHG4, and AP003392.5. SNHG4 has been extensively studied in several cancers. Long Non-Coding RNA SNHG4 was a biomarker in Non-Small Cell Lung Cancer in colorectal cancer (34, 35). However, there are not enough studies on the effect of SNHG4 on liver cancer, and we used a cell assay to analyze the impact of SNHG4 in liver cell carcinoma. Clone formation experiments showed that the number of clones formed by Hep G2 and Hep-3b cell lines in the si1-SNHG4 and si2-SNHG4 groups after culture and staining was significantly lower than that of the si-NC group (P < 0.05) **(** **Figures 8A**, **B** **).**

### Transwell Assay

Compared with the si-NC control group, the number of Hep-G2 and Hep-3b cells passing through the Transwell chamber in the Si1-SNHG4 and si2-SNHG4 groups was significantly lower, suggesting SNHG4 promotes the migration of Hep-G2 and Hep-3b cells (P < 0.05) **(** **Figures 8C**, **D** **)**.

### Knocking SNHG4 Affects Wnt Signaling Pathways

**Figure 9:**
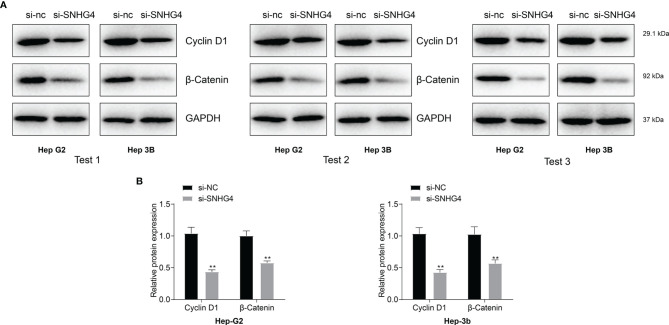
**(A)** Protein levels of WNT signaling pathway-related molecules (β-Catenin and cyclin D1) were measured using western blotting in HEP-G2 and HEP-3B cell lines. **(B)** Knocking down SNHG4 down-regulated the WNT signaling pathway and affected the expression of cyclin D1. * represents p < 0.05; ** represents p < 0.01; *** represents p < 0.001.

After pathway enrichment analysis in the TCGA-LIHC cohort, SNHG4 was found to be closely associated with liver cancer progression. To verify the specific effect mechanism of SNHG4 on liver cancer, hepatocyte carcinoma cell lines of HEP-G2 and HEP-3B cells with SNHG4 knockdown cell lines were used for Western blotting analysis. The results showed that the expression level of cyclin D1 and β -catenin protein in the SNHG4 knockout group was significantly lower than that in the negative control group (NC) **(** **Figures 9A, B** **).** Three repeated experiments demonstrated that knocking down SNHG4 down-regulated the WNT signaling pathway and affected the expression of cyclin D1.

## Discussion

The mechanism of m5C methylation modification of lncRNA is unclear; therefore, we attempted to comprehensively analyze lncRNA related to m5C methylation modification using computational biology. The m5C methylation modification of RNA is dynamically regulated by methyltransferase and demethyltransferase. Under the action of methyltransferase, RNA undergoes m5C modification and then combines with recognition protein to exert specific biological functions (3). The methyltransferases modified by m5C include NSUN1, NSUN2, NSUN3, NSUN4, NSUN5, NSUN7, and DNMT2. The point is a structurally conserved cysteine residue that catalyzes m5C methylation in various types of RNA with the help of the methylated donor S-adenosine-L-methionine. Therefore, we focused on the lncRNA, regulated by the aforementioned transferases.

It is believed that m5C methyltransferase regulates lncRNA in liver cancer. The role of m5C methylation in the occurrence and progression of cancer has been identified in liver cancer, including mRNA, microRNA, lncRNA, and other types of RNA. The m5C modification of RNA plays an essential regulatory role in the occurrence and progression of tumors (13). The m5C methyltransferase NSUN4 recognizes protein ALYREF associated with liver cancer outcomes. A study found that expression levels of H19 lncRNA in cancer tissues were significantly higher than those in adjacent tissues. Other studies found that this effect was due to the m5C modification on H19 lncRNA mediated by NSUN2, which increases the stability of H19 lncRNA. H19 lncRNA with m5C change specifically binds to G3BP1 protein, further leading to the accumulation of oncoprotein and promoting the occurrence and progression of liver cancer (36).

We identified SNHG4 as an m5C methylation modification lncRNA in the present study. SNHG4 encodes small nucleolar RNA host gene 4. Some lncRNAs encode small nuclear RNA host genes. In recent years, many studies have found that the abnormal expression of snRNAs may play the role of oncogenes in the development of tumors. For example, Chen found that SNHG8 was upregulated in non-small cell lung cancer (37). Other investigators found that the expression trend of SNHG8 in glioma and liver cancer was consistent with these studies (38–40).

There are few in-depth studies discussing the predictive value of SNHGs. SNHG1, SNHG3, and SNHG20 are predictive biomarkers for neuroblastoma (41), ovarian cancer (42), and colorectal cancer (43), respectively. Zhu et al. conducted a bioinformatics analysis of lncRNA and found that SNHG4 may be a valuable prognostic marker in HCC (40). In the present study, we reached the same conclusion that the expression of SNHG4 was an independent predictor of poor outcomes in HCC. We further studied the predictive value of SNHG4 in the subgroups and found its limitations in women and young patients, which may help direct precision therapy.

There are some limitations to our paper. We only analyzed lncRNAs associated with m5C transferase in TCGA; more sequencing cohorts are needed to validate our findings. This paper only conducted a comprehensive analysis of m5C related lncRNA and did not include a complete regulatory mechanism study.

## Conclusion

We immediately identified 436 m5C transferase-related long non-coding RNAs and 43 prognostic-related lncRNAs related to m5C transferase. Four lncRNA were determined by LASSO regression to reduce the screening range further. Finally, we found that SNHG4 was significantly associated with the protein-coding gene of m5C methyltransferase. Cell experiments showed that knocking down SNHG4 affected the proliferation and migration of HCC. This comprehensive analysis of lncRNA regulated by m5C transferase provides a basis for future research on the methylation regulation of long-chain non-coding RNA.

## Data Availability Statement

Publicly available datasets were analyzed in this study. This data can be found here: The datasets TCGA-LIHC for this study can be found at http://cancergenome.nih.gov/.

## Author Contributions

QP wrote the manuscript. CY and YZ revised the manuscript. All authors contributed to the article and approved the submitted version.

## Conflict of Interest

The authors declare that the research was conducted in the absence of any commercial or financial relationships that could be construed as a potential conflict of interest.

## Publisher’s Note

All claims expressed in this article are solely those of the authors and do not necessarily represent those of their affiliated organizations, or those of the publisher, the editors and the reviewers. Any product that may be evaluated in this article, or claim that may be made by its manufacturer, is not guaranteed or endorsed by the publisher.

## Abbreviations

LIHC, Liver hepatocellular carcinoma; M5C, 5-Methylcytosine RNA methyltransferases; NMF, non-negative matrix factorization; WGCNA, Weighted correlation network analysis; LASSO, Least absolute shrinkage and selection operator; GSEA, Gene Set Enrichment Analysis; ESTIMATE, Estimation of Stromal and Immune cells in Malignant Tumor tissues using Expression data; SSGSEA, single sample gene set enrichment analysis.
